# Tribo-electrification and Powder Adhesion Studies in the Development of Polymeric Hydrophilic Drug Matrices

**DOI:** 10.3390/ma8041482

**Published:** 2015-03-30

**Authors:** Muhammad U. Ghori, Enes Šupuk, Barbara R. Conway

**Affiliations:** 1 Department of Pharmacy, University of Huddersfield, Huddersfield HD1 3DH, UK; E-Mails: m.ghori@hud.ac.uk (M.U.G.); e.supuk@hud.ac.uk (E.S.); 2 Department of Chemical Sciences, University of Huddersfield, Huddersfield HD1 3DH, UK

**Keywords:** tribo-electric charging, hydroxypropyl methylcellulose, methylcellulose, surface adhesion, theophylline

## Abstract

The generation of tribo-electric charge during pharmaceutical powder processing can cause a range of complications, including segregation of components leading to content uniformity and particle surface adhesion. This phenomenon becomes problematical when excipients are introduced to a powder mixture alongside the highly charging active pharmaceutical ingredient(s) (APIs). The aim of this study was to investigate the tribo-electric charging and adhesion properties of a model drug, theophylline. Moreover, binary powder mixtures of theophylline with methylcellulose (MC) and hydroxypropyl methylcellulose (HPMC), having different polymer to drug ratios, were formed in order to study the impact of polymer concentration, particle size, substitution ratio and molecular size on the tribo-electric charging and surface adhesion properties of the drug. Furthermore, the relationship between tribo-electric charging and surface adhesion was also studied. The diversity in physicochemical properties of MC/HPMC has shown a significant impact on the tribo-electric charging and adhesion behaviour of theophylline. It was found that the magnitude of electrostatic charge and the level of surface adhesion of the API were significantly reduced with an increase in MC and HPMC concentration, substitution ratios and molecular size. In addition, the tribo-electric charge showed a linear relationship with particle surface adhesion, but the involvement of other forces cannot be neglected.

## 1. Introduction

The pharmaceutical industry relies heavily on powder processing, since more than 80% of its products are provided in tablet form. In order to manufacture a tablet, the excipients and active pharmaceutical ingredient (API) need to be mixed together thoroughly to form a homogenous powder mixture [1]. The pharmaceutical industry is heavily regulated with products having specific quality control requirements regarding uniformity of active contents, consistency in appearance, longevity for storage, transportation and shelf life, demanding an exceptional degree of control and precision [2,3]. However, during powder processing, powder particles frequently come into contact with each other and with the walls of the processing equipment leading to the generation of tribo-electric charge on the surface of powder particles [4]. This phenomenon becomes more significant when multiple powder formulations containing excipients and APIs are mixed together as particulate interactions may give rise to electrification and surface adhesion of powder particles. Therefore, the study and characterisation of electrostatic and surface adhesion properties of powders are vitally important in the development of tablet dosage forms [5,6].

There are three fundamental mechanisms (electron transfer, ion transfer and material transfer) that contribute to the resulting charge that is generated by the tribo-electric process. However, electron transfer is considered to be the main mechanism for pharmaceutical insulating materials. During the contact charging process, the valence electron energy state of powder particles on an atomic scale is designated as the fermi level whereas the vacuum energy level is a thermodynamic state of electrons far from the atom and can be considered as a reference point. The difference between the fermi level and vacuum energy level equates to the work function (∅), which is a unique surface property of materials and refers to the minimum energy difference required for the liberation of loosely bonded electrons present in the outer electron shells of an atom when inter- or intra-particulate contacts of powder particles are established. As a consequence, the electrons flow from the lower work function ($\emptyset_{1}$) towards the higher ($\emptyset_{2}$), thus a potential difference is generated across the particle surface [7]. The charge transferred (*Q*) during contact can be described using Equation (1) [8];

(1) $$Q=CV_{c}=C\frac{\left(\emptyset_{1}-\emptyset_{2}\right)}{e}$$

where *C* is the capacitance; e is the charge on an electron; *V*_c_ is the contact potential difference; and ∅_1_ and ∅_2_ are work function of material being tested and reference material, respectively. The capacitance (*C*), which is the ability to store electrical energy as its potential rises, is given by Equation (2) [8];

(2) $$C=\frac{\varepsilon_{0}\;S}{z}$$

with *S* being the effective area of contact; *z* the separation at contact; and ε_0_ the permittivity of free space.

The electrostatic particle charging is a common problem as it can cause segregation, dust explosions, adhesion and deposition or blockage of pipelines, leading to loss of powder and difficulties controlling the powder flow [6,7,8,9,10]. Therefore, the ability to control the charging of pharmaceutical powders is considered essential in improving the quality of the end product and can minimise deposition and powder loss. Thus powder-processing techniques used require in depth evaluation and further adaptation to meet these challenges. In spite of the negative influences described above, electrostatic charging phenomena can be beneficial under certain conditions, for example, engaging the opposite polarity of charged powder particles in order to fabricate ordered mixtures. Moreover, electrostatic assisted ordered mixtures are considered to be more consistent and have the potential to improve content homogeneity, stability and address powder processing problems [11,12]. A wide range of pharmaceutical excipients is employed to develop compressed hydrophilic matrices, acting as binders, drug release modifiers, lubricants and disintegrants in order to improve the processability of the formulation and bioavailability of the drug. However, hydrophilic derivatives of cellulose ethers, more specifically methylcellulose (MC) and hydroxypropyl methylcellulose (HPMC), are considered to be the main excipients of choice in the development of compressed hydrophilic matrix tablets [13,14]. Recently, tribo-electric charging behaviour of Methocel^®^ A4M, F4M, E4M, K4M, K15M and K100M, in plain and binary mixtures with the negatively charging API, flurbiprofen, have been studied [15]. The magnitude of the positive charge of the polymers, was distinct from the majority of other excipients previously reported, and was generally higher [9,15]. Furthermore, it was reported that when highly charged flurbiprofen came into contact with Methocel^®^, irrespective of its grade, it attached to its surface due to opposite polarities and the tribo-electric charge of the final powder mixture was significantly dissipated [15]. Moreover, Asare-Addo *et al.* (2013) [16] described the tribo-electric charging behaviour of Methocel^®^ E4M, K4M and their powder mixtures with the negatively charging API, theophylline. It showed that when theophylline came into contact with HPMC, it attached to its surface due to having opposite polarity and the tribo-electric charge of the final powder mixture was decreased. However, the impact of diverse physico-chemical attributes related to MC and HPMC on API in a binary system still needs further investigation. Therefore, the aim of this present study was to investigate the tribo-electrification and adhesion properties of different MC and HPMC based powder mixtures, using theophylline as a model drug. The impact of polymer attributes [concentration, particle size, hydroxypropyl (Hpo)/methoxyl (Meo) substitution ratio and molecular size] on tribo-electric charging and surface adhesion of powder mixtures were studied. Furthermore, a relationship between tribo-electric charging and surface adhesion was also investigated.

## 2. Results and Discussion

### 2.1. Characterisation of Powders

**Figure 1:**
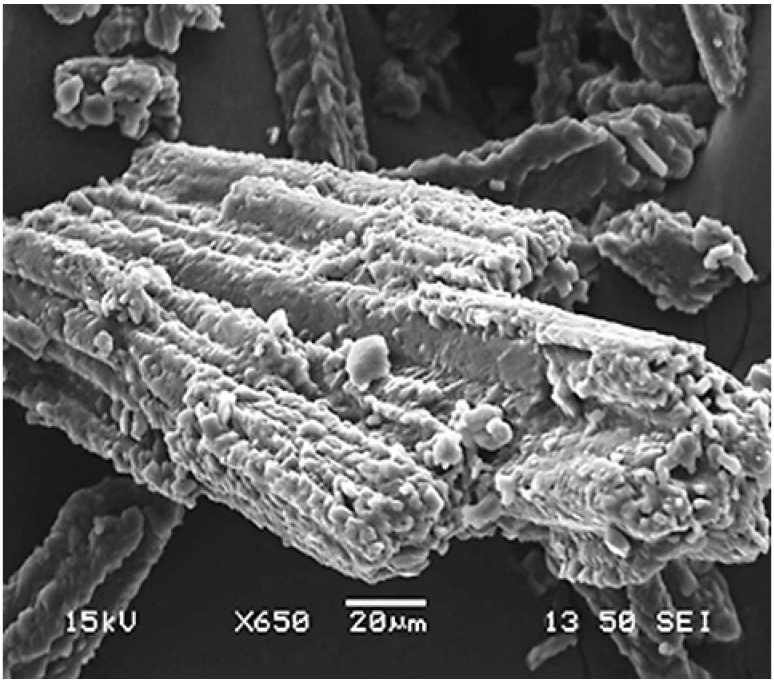
Scanning electron microscopy (SEM) micrograph of theophylline powder particles.

SEM micrographs were used to study the surface morphology of pure model drugs. Figure 1 illustrates SEM micrograph of theophylline (THP), which was imaged at a magnification of ×650 and it has elongated crystals with a columnar habit. From the SEM micrographs, it can be concluded that the crystals have a rough surface. Moreover, all the grades of HPMC and MC contained mixtures of irregular-shaped flat and fibrous particles (supplementary data Figure S1a–f). Generally, the proportion of fibrous material is higher in MC than HPMC. The K-chemistry grades of HPMC and, in particular, K100M contain more irregularly shaped particles with rough surfaces than any of the other grades of cellulose ethers. This is attributed to the higher Hpo/Meo substitution ratio and molecular size, which result in more complex surfaces [15,17].

### 2.2. Tribo-Electric Charging and Surface Adhesion Properties of Theophylline

The model drug, THP, like many other APIs, possesses a high degree of crystallinity and would be presumed to have a high electrical resistance [18]. THP powder particles attained maximum level of charge (−32 nC/g, Table 1), Q_max_, in 2 min when shaken inside a stainless steel container.

**Table 1:** Tribo-electric charging and surface adhesion of THP (*n* = 3, standard deviations in parentheses).

Drug	Particle size	Charge to mass ratio (nC/g)	Adhesion (%)
Theophylline (THP)	38–63 μm	−32.01 (3.8)	38.42 (5.11)

**Figure 2:**
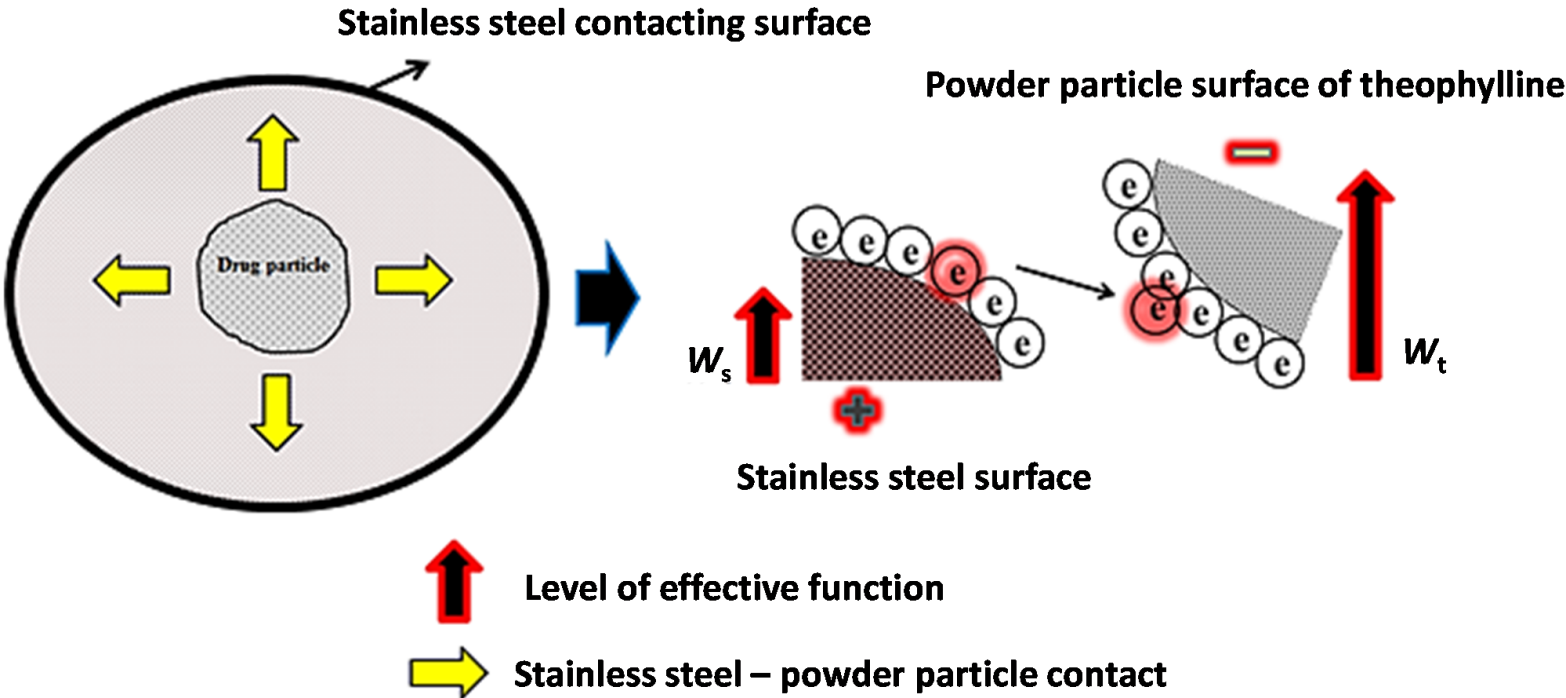
Schematic illustration of tribo-electric charging of theophylline (THP) powder particles.

The tribo-electric charge generation is considered to be ambiguous, but the electron transfer theory is widely accepted to explain the mechanism of charge generation of contacting surfaces. The effective work function of theophylline (*W_t_*) is likely to be higher than the work function of stainless steel surface (*W_s_*) hence becoming negatively charged (as illustrated in Figure 2). The work function of a theophylline depends on its physical and chemical properties, including environmental conditions. Electrostatic forces on theophylline particles arise primarily from the presence of excess electric charge on the particles. A charged particle near a conductive surface will be affected by the image force due to induced image charges [19]. The image force attracts particles toward the surface and act as an adhesive. The results show THP was inclined to have lower surface adhesion, which might be due to the fact that lower levels of tribo-electric charging tend to generate weak inter- or intra-particulate dispersive forces. Hence, the electrostatic behaviour of THP can be categorised as a low charging when compared to other APIs [9,14].

### 2.3. Characterisation of Powder Blends

**Figure 3:**
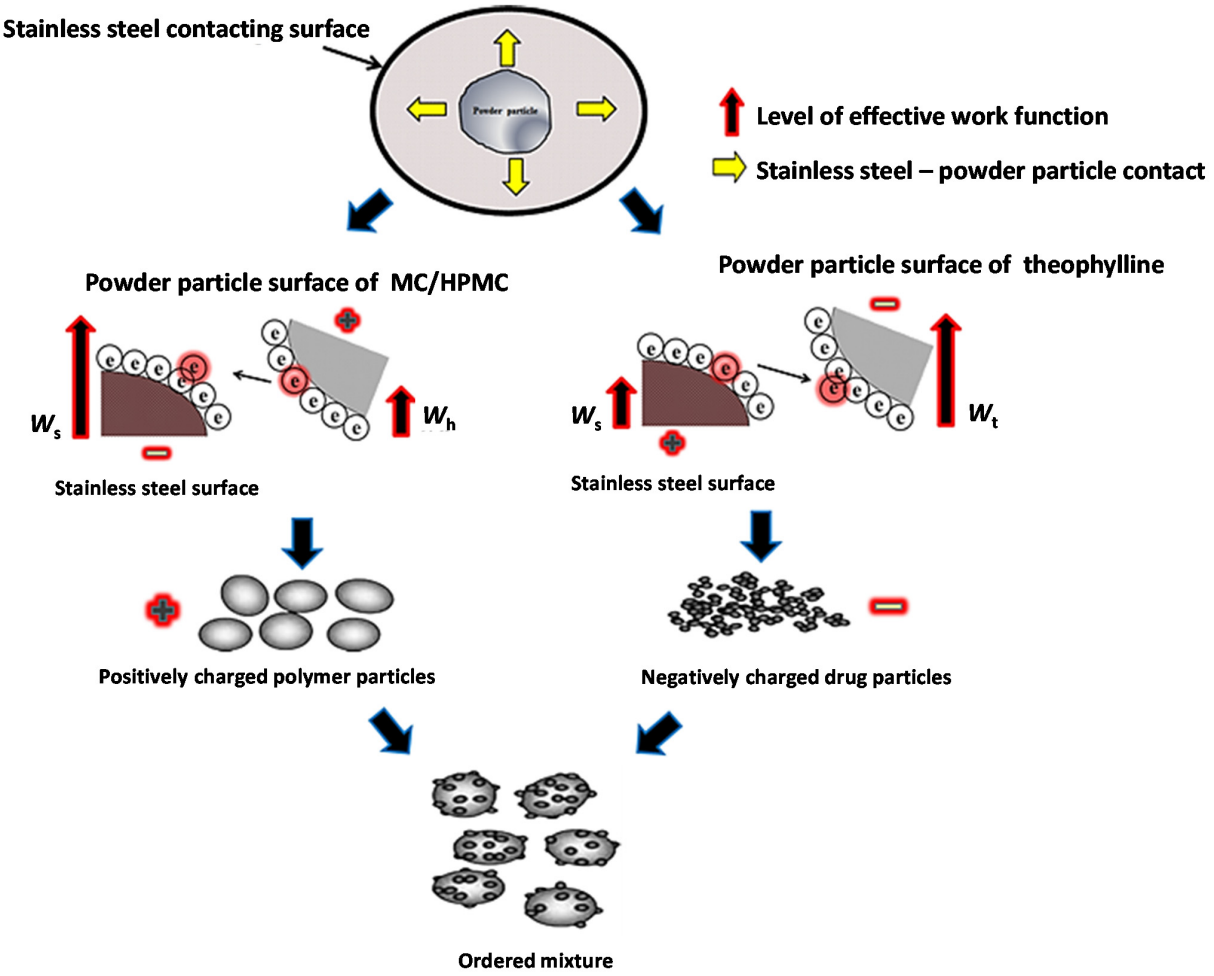
Schematic illustration of electrostatic assisted powder mixtures.

**Figure 4:**
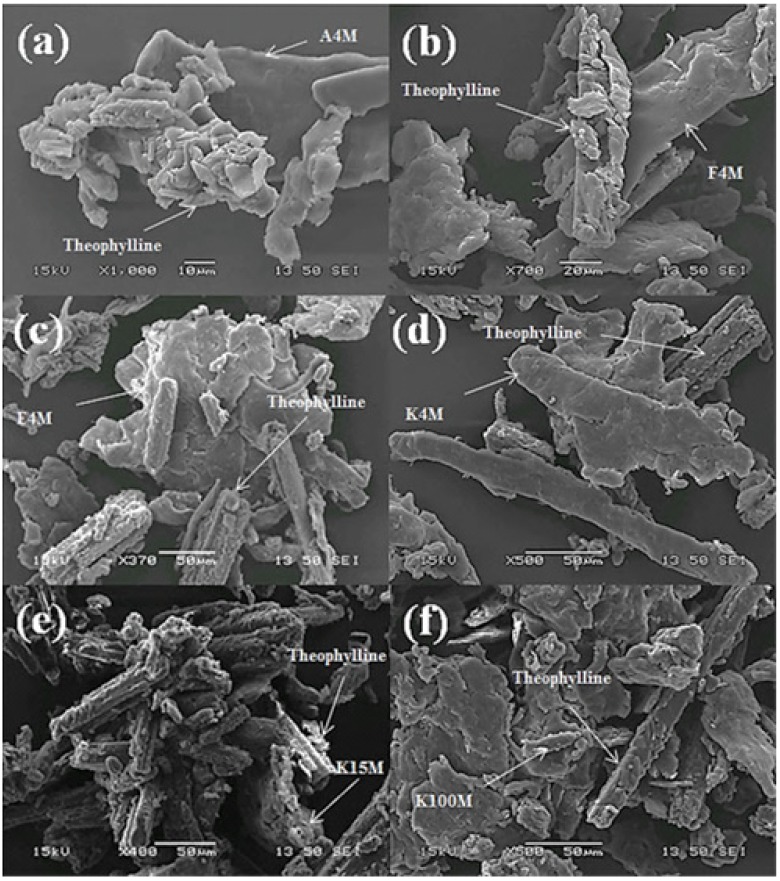
SEM micrographs of THP powder mixtures, (**a**) A4M/THP; (**b**) F4M/THP; (**c**) E4M/THP; (**d**) K4M/THP; (**e**) K15M/THP; and (**f**) K100M/THP.

Among different mixing methods, ordered mixing results in more homogeneous and stable particulate powder mixtures. Ordered mixing can be achieved using powder particles having electrostatic charges of opposite polarity [11,19]. In most cases, drugs are negatively charged while the excipients or polymers are positively charged. This can be used to maintain the homogeneity of a mix through different processing conditions [19,20,21]. In the present study, an ordered binary mix was formed by mixing a polymer (positively charged) and THP (negatively charged) powders. The powder particles of polymers and drugs gained positive and negative tribo-electric charge during tribo-electrification with a stainless steel surface, respectively, due to the difference in the work functions between the powder particles and contacting surfaces. In the present study, when THP charged negatively and polymers charged positively, it was observed by using scanning electron (SEM) that the negatively charged THP powder particles were attracted towards the positively charged polymeric powder particles and adhered to their surfaces. A schematic illustration of powder charging and the attachment of fine drug particle to the polymer surface are shown in Figure 3. Pharmaceutical formulations have stringent quality control requirements with regard to drug content uniformity, so all binary mixtures were analysed and contained between 95% and 105% of the expected drug (THP) content. To validate the attachment of negatively charged drug particles on the positively charged polymer particle and further confirm the formation of electrostatic ordered powder mixtures, SEM was used. The general findings from this study are exemplified in Figure 4, which illustrates six SEM micrographs of THP powder mixtures with different grades of MC and HPMC. The negatively charged THP powder particles are attached to the positively charged polymer surface, regardless of grade. Furthermore, it is clearly evident from the micrographs that the THP has retained its crystalline habit. Overall, it can be concluded from the SEM images that the negatively charged THP powder particles are attached to the positively charged MC or HPMC powder particles and this leads to the successful formation of homogeneous, electrostatic-assisted, ordered mixtures.

**Figure 5:**
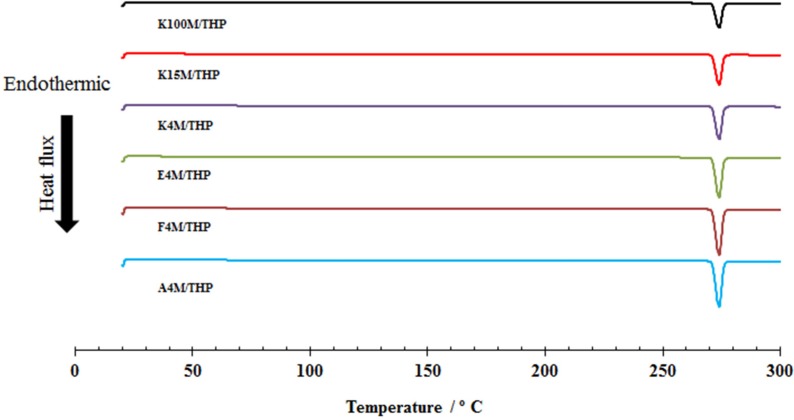
Differential scanning calorimetry (DSC) profiles of theophylline powder mixtures.

DSC was used to investigate any possible solid-solid interaction between THP and MC/HPMC. Figure S2 (supplementary data) depicts the DSC thermograms of MC and HPMC showing no melting peak, suggesting they are amorphous. However, Figure S3 (supplementary data) demonstrated sharp endothermic melting peaks at 272.02 °C for THP. Moreover, Figure 5 shows DSC thermograms of MC/HPMC:THP powder mixtures containing 15 % w/w of polymer content to exemplify and indicate any possible drug-polymer interaction. There was a negligible depression in the melting peaks and it can be concluded that the drugs retained their crystalline structure and no drug-polymer interaction was discerned.

**Figure 6:**
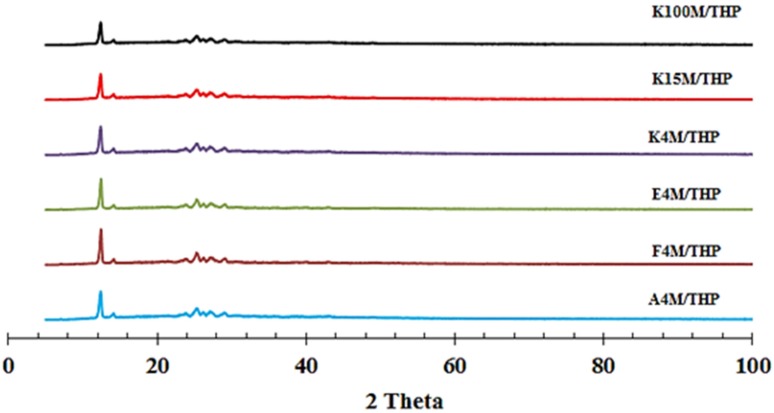
X-ray diffraction (XRD) profiles of theophylline powder mixtures.

Figure S4 (supplementary data) shows the characteristic XRD diffraction peaks at 12.30° and 25.22° for THP. The sharp and intense diffraction peaks of model drug reflect its crystalline structure. Figure 6 shows XRD patterns of MC/HPMC:THP powder mixtures containing 15 % w/w of polymer contents to exemplify and indicate any possible drug-polymer interaction. It is evident that the THP diffraction pattern was unchanged, which confirms that after powder mixing, the drug retained its crystalline structure. Furthermore, it can be concluded that no drug-polymer interaction was evident.

### 2.4. Tribo-Electric Charging and Surface Adhesion Properties of Powder Blends

#### 2.4.1. Effect of MC/HPMC Concentration

**Figure 7:**
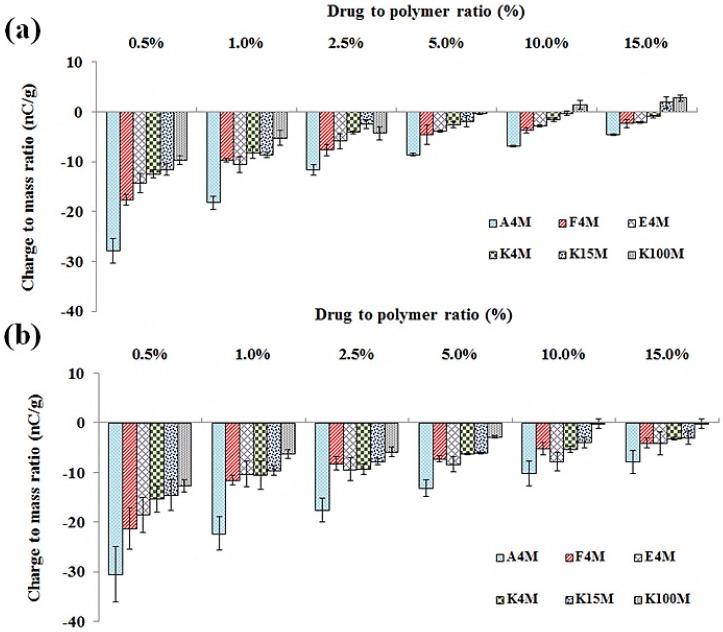
Effect of polymer concentration on the tribo-electric charging of cellulose ether: THP powder mixtures; polymer particle size (**a**) 150–250 μm; and (**b**) 90–150 μm (*n* = 3).

The acquisition of tribo-electric charge and surface adhesion of powder formulations can be affected by the component ratios in a binary powder mixture [15,22,23]. Tribo-electric charging and adhesion experiments were carried out on MC/HPMC:THP powder mixtures with fixed polymer to drug loading ratios of 0.5, 1, 2.5, 5, 10 and 15 % w/w. The charging and adhesion results show that the addition of increasing proportions of MC/HPMC has a significant impact (level of estimated probability (*p* < 0.05)) on these properties; as the level of MC/HPMC increased from 0.5% to 15%, the charge and powder surface adhesion was decreased (Figure 7a,b and Table 2).

In case of MC/HPMC:THP powder mixtures, at 0.5% polymer content, there is a slight decrease in the charge and powder surface adhesion of THP with the addition of A4M, F4M and E4M. With further increases in polymer concentration, the charge dissipation increases significantly due to the influence of the polymer-to-drug ratio on the overall effective work function and surface resistivity of a bulk powder sample (Figure 7a,b and Table 2). The charge reduction reached a plateau level at 5% and 10% and so further increases in polymer concentration only had a small impact on charge; however, the powder surface adhesion was further reduced. The powder mixtures of THP formed using polymers K4M, K15M and K100M were lower charging than A4M, F4M and E4M. The addition of 0.5 % w/w of polymer halved the overall net charge on THP. Moreover, at 15% polymer, the charge was neutralised completely and particle adhesion was also very low. For the powder mixtures, at 15% polymer content, significant dissipation of the tribo-electric charge was noticed and this phenomenon was previously discussed [16], however in that study 20 % w/w of E4M and K4M with THP was used. The charge reduction is likely due to ordering of particles as particles of opposite charges adhere to each other as electrovalent bonds develop between drug and excipient powder particles due to an exchange of electrons.

**Table 2:** Powder surface adhesion (%) of polymer/THP powder mixtures for different particle size fractions (standard deviation in parentheses, *n* = 3).

Methocel^®^	Particle size (μm)	Surface adhesion (%)					
		Methocel^®^ concentration (%)					
		0.5	1	2.5	5	10	15
A4M	**90–150**	35.1 (5.27)	30.2 (4.53)	28.1 (5.63)	22.2 (4.89)	20.3 (4.69)	18.5 (4.64)
	**150–250**	34.6 (5.20)	28.3 (4.25)	20.5 (4.12)	18.6 (4.11)	15.1 (3.47)	12.6 (3.17)
F4M	**90–150**	33.8 (5.08)	27.5 (4.14)	22.5 (4.51)	15.5 (3.43)	17.3 (3.99)	14.2 (3.57)
	**150–250**	32.2 (4.84)	21.5 (3.24)	16.2 (3.25)	11.4 (2.52)	10.7 (2.46)	8.8 (2.22)
E4M	**90–150**	30.8 (4.62)	21.3 (3.20)	16.3 (3.27)	13.9 (3.08)	10.5 (2.43)	9.5 (2.40)
	**150–250**	27.3 (4.11)	18.2 (3.29)	14.3 (2.87)	11.4 (2.51)	8.5 (1.97)	7.5 (1.89)
K4M	**90–150**	27.3 (5.47)	17.5 (3.15)	14.3 (2.88)	9.6 (2.13)	8.9 (2.06)	6.2 (1.55)
	**150–250**	20.7 (4.16)	11.5 (2.07)	9.7 (1.95)	7.6 (1.69)	7.1 (1.64)	5.5 (1.40)
K15M	**90–150**	24.3 (4.88)	18.6 (3.36)	11.3 (2.26)	8.8 (1.95)	7.5 (1.74)	5.8 (1.47)
	**150–250**	14.3 (2.88)	9.6 (1.74)	7.3 (1.46)	6.8 (1.71)	4.5 (1.05)	3.8 (0.97)
K100M	**90–150**	18.3 (3.66)	9.5 (1.71)	7.2 (1.44)	6.2 (1.56)	5.8 (1.35)	4.1 (1.05)
	**150–250**	8.3 (1.66)	6.5 (1.17)	5.2 (1.04)	3.9 (1.00)	3.8 (0.89)	2.90 (0.75)

**Figure 8:**
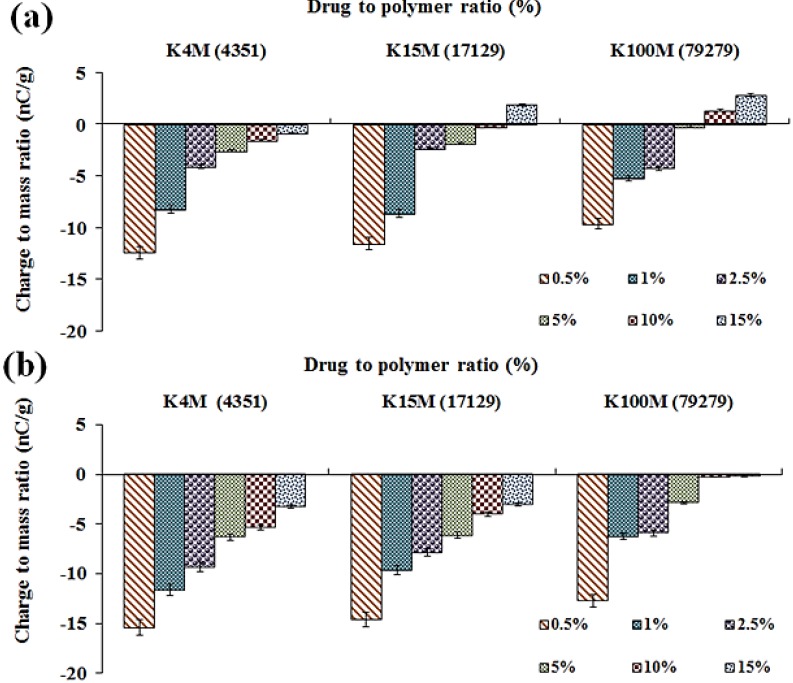
Effect of polymer molecular size (viscosity) on the tribo-electric charging of MC/HPMC:THP powder mixtures; polymer particle size (**a**) 150–250 μm; and (**b**) 90–150 μm, *n* = 3, (viscosity (cps) in parentheses on x-axis).

Additionally, in the present study, a shift in polarity of charge from negative to positive was also evident for THP powder mixtures at 15 % w/w concentrations of K15M and K100M. Additionally, K100M also showed a shift in polarity at 10 % w/w concentration (Figure 8). This phenomenon was previously encountered for glucose/lactose [23] and Methocel^®^:flurbiprofen powder mixtures [15]. In the existing situation, it can be anticipated that the K15M and K100M have a lower work function and surface resistivity than THP and the contacting surface (stainless steel). Thus, when the percentage of polymer is increased, the net surface resistivity and effective work function of powders is altered, leading to a reduction and shift in polarity of electrostatic charge. As expected, such a significant charge reduction changes the classification from a higher charging to a lower charging category, and the impact of charge during powder handling will be reduced.

#### 2.4.2. Effect of MC/HPMC Particle Size

The effect of polymer particle size on the propensity of charge and surface adhesion of THP powder mixtures during the contact electrification process against a stainless steel surface is shown in Figure 7a,b and Table 2, respectively. Charge and surface adhesion of all the powder mixtures were increased as the particle size of MC/HPMC was reduced from 150–250 to 90–150 μm, regardless of concentration and MC/HPMC grade. It was further noticed that the larger particle size (150–250 μm) of K15M (15% polymer) and K100M (10% and 15% polymer) caused a shift in polarity of charge from negative to positive. This particular phenomenon was previously reported by our group, and with the theory proposed by Gallo and Lama [24] in which it was postulated that the work function decreases with an increase in particle size and the electrons always move from a material having lower work function, that is, it acted as a donor eventually gaining a positive charge. This particle size alteration and ordering of oppositely charged powder particles reduce the electrostatic charge and surface adhesion of powder particles. Furthermore, the particle size variation might also change the effective work function of powder blends as previously explained by various authors [12,15,19,23,24]. So, the findings of the current study imply that the manipulation of polymer particle size may aid reduction of electrostatic properties and adhesion of pharmaceutical powder mixtures.

#### 2.4.3. Effect of MC/HPMC Degree of Substitution

The chemistry of polymers can affect the tribo-electric charging and surface adhesion properties of powders as it instigates variations in particle dynamics during the tribo-charging process. The major difference between MC (A4M) and other various grades of HPMC (F4M, E4M and K4M) is the levels of Meo and Hpo groups attached to the parent glucose ring. The variation in the Hpo/Meo substitution ratios had a significant impact on the tribo-electric charge and surface adhesion properties of plain MC and HPMC powders [15]. In the present study of MC/HPMC:THP powder mixtures, the Hpo to Meo substitution ratio had a significant impact on behaviour tribo-electric charging and surface adhesion. It is notable that as the substitution ratio increases, the surface irregularities were increased, resulting in a more complex surface morphology of powder particles. The separation difference, which also affects the charge transfer will be different, hence affect the magnitude of charge, as increasing the separation difference will decrease charge transfer [8]. The overall net charge and surface adhesion were decreased with an increase in Hpo/Meo substitution ratios (*i.e.*, A > F > E > K) and this was the case for both particle size fractions (Figure 8 and Table 2).

#### 2.4.4. Effect of MC/HPMC Molecular Size (Viscosity)

The molecular size of MC/HPMC can affect charging and surface adhesion due to its impact on the polymer chain lengths and their subsequent packing in a powder particle. The molecular size of MC/HPMC has a significant effect on the tribo-electric charging and surface adhesion properties of powder mixtures containing THP (Figure 8a,b and Table 2). The net tribo-electric charge and surface adhesion were decreased with an increase in the molecular size (for the K series); both the particle size fractions showed similar behaviour. It is evident that as molecular size increases, it affects the packing of the polymeric chains, which leads to irregularities on the surface powder particles, as previously described by our group in the recent publication [15]. These changes can modify surface resistivity and the effective work function of powder particles in a blend. Moreover, these characteristics dictate the charge transfer process and generation of operational forces (van der Waals forces, ionic bonding and electrostatic forces), thus impacting tribo-electrification phenomena. Additionally, it can be presumed that the agglomeration and stability of these powder mixtures can be improved with more complex surface carrier characteristics compared with particles presenting a smooth surface [19,21].

### 2.5. Relationship between Tribo-Electric Charge and Powder Surface Adhesion

**Figure 9:**
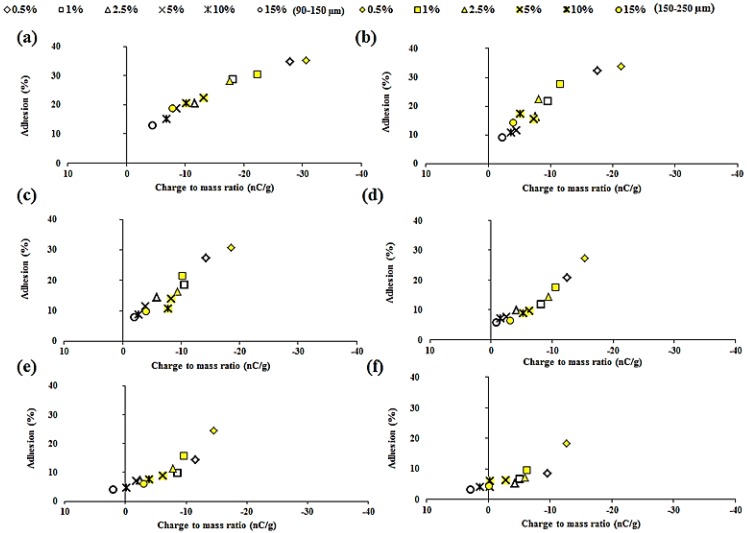
Effect of tribo-electric charging (nC/g) of THP powder mixtures on surface adhesion (SA) (%), (**a**) A4M; (**b**) F4M; (**c**) E4M; (**d**) K4M; (**e**) K15M; and (**f**) K100M having polymer particle size 90–150 μm and 150–250 μm.

Table 2 shows the adhesion of polymer particles to the steel surface, calculated as a ratio between the initial feed and the mass loss due to powder sticking. A reduction in adhesion is observed with a decrease in electrostatic charge on the particles. Figure 9 shows the relationship between charge and surface adhesion for all binary mixtures having varying polymer particle size (90–150 μm and 150–250 μm) and concentration (0.5–15 % w/w). A reduction in susceptibility towards tribo-electric charging is directly related to surface adhesion, with correlation coefficients ranging between 0.750 and 0.99 (Table 3). The F4M:THP (particle size 150–250 μm) blends showed a relatively strong correlation, but correlation coefficients of A4M powder mixtures had lower values. The particle size fraction also has a significant impact on the R^2^, which tends to decrease with decreasing particle size. The current study shows that electrostatic forces generated during the tribo-electrification process played a significant role during the surface adhesion phenomena of pharmaceutical powders processing. However, it is appreciated that the mechanism of particle adhesion is a multifaceted process and other mechanisms may also be involved [15,25,26].

**Table 3:** Inter-relationship between tribo-electric charge and surface adhesion (*n* = 3).

Powder mixtures	Correlation co-efficient (R^2^)	
	Particle size (90–150 μm)	Particle size (150–250 μm)
A4M/THP	0.750	0.947
F4M/THP	0.880	0.992
E4M/THP	0.917	0.967
K4M/THP	0.982	0.930
K15M/THP	0.975	0.938
K100M/THP	0.899	0.963

## 3. Experimental

### 3.1. Materials

Theophylline was purchased from Tokyo Chemical Industry Ltd., Oxford, UK. Methylcellulose, (Methocel^®^ A4M) and hypromellose (Methocel^®^ F4M, E4M, K4M, K15M and K100M) were kindly donated by Colorcon Ltd. (Dartford, UK) and specifications are listed in Table 4.

### 3.2. Methods

#### 3.2.1. Powder preparation and characterisation

Particle size fractions of each polymer (90–150 μm and 150–250 μm) and theophylline (38–63 μm) were obtained through mechanical sieving. All the powders were stored at ambient temperature (18–24 °C) and humidity (RH 36%–44%) before any further investigations. Surface morphology was imaged using scanning electron microscopy (SEM). All samples were sputter-coated with gold/palladium (80:20) for 60 s using the Quorum SC7620 Sputter Coater and samples imaged using the Jeol JSM-6060CV SEM (JEOL USA, Inc., Peabody, MA, USA) under vacuum.

**Table 4:** Specifications of methylcellulose (MC) and hypromellose (HPMC).

Methocel^®^ grade	Methoxy (Meo) (% w/w) ^a^	Hydroxypropyl (Hpo) (% w/w) ^a^	Hpo/Meo ratio	Total substitution (% w/w)	Viscosity (cps) ^a^
A4M	30	0	0	30	4878
F4M	28.1	6.7	0.238	34.8	4031
E4M	29.0	8.3	0.286	37.3	3919
K4M	22.3	8.5	0.381	30.8	4351
K15M	22.3	9.0	0.403	31.3	17129
K100M	22.5	8.9	0.395	31.4	79279

^a^ Data obtained from the manufacturer.

#### 3.2.2. Preparation and Storage of Powder Mixtures

Binary powder blends of theophylline (38–63 μm) and the cellulose ethers (90–150 μm and 150–250 μm size fractions) were prepared as described in Table 2, at a fixed polymer to drug ratio of 0.5, 1, 2.5, 5, 10 and 15 % w/w. The powder samples were tumble mixed for 20 min (50 rpm) and stored at an ambient temperature (18–24 °C) and humidity (RH 36%–44%).

#### 3.2.3. Efficiency of Mixing

##### Content Uniformity of Powder Blends

To ensure a homogeneous powder mixture was achieved, random samples of 10 mg were taken from each batch (*n* = 3) and dissolved in 100 mL of pH 7.2 phosphate buffer. As the complete powder contents were dissolved, a 5 mL aliquot was extracted using plastic syringe fitted with 0.45 μm PTFE syringe filter. The concentration of theophylline contents was determined by using UV-Vis Spectrophotometry (Jenway 6305, UV-VIS spectrophotometer, Bibby Scientific Ltd., Staffordshire, UK, λ_max_ = 272 nm) and an acceptance limit of 95%–105% was set [27].

##### Differential Scanning Calorimetry (DSC) of Powders

In this study DSC (Mettler Toledo SC 821, Mettler-Toledo Ltd., Leicester, UK) analysis for all the powder samples (plain drug, polymers and their respective powder mixtures) was performed using 5–10 mg of powder samples in an atmosphere of flowing nitrogen at 50 mL per minute and temperature program of 10 °C/min from 20 °C to 300 °C.

##### X-ray Diffraction (XRD) of Powders

In this study, the powder X-ray diffraction study of all the powder samples (plain drug, polymers and their respective powder mixtures) was carried out using a D2-Phase X-ray diffractometer (Bruker UK Ltd., Coventry, UK) equipped with a CuKɑ radiation source at 30 KV voltage and 10 mA current. Diffraction patterns were obtained in the 2θ range of 5°–100° using 0.02 step sizes.

##### Scanning Electron Microscopy (SEM) of Powder Blends

The morphology of individual particles and attachment of drug particles to the surface of polymer particles was observed using SEM. All samples of theophylline powder blends were sputter-coated with gold/palladium (80:20) for 60 s using the Quorum SC7620 Sputter Coater and samples imaged using the Jeol JSM-6060CV SEM (JEOL USA, Inc., Peabody, MA, USA) under vacuum.

#### 3.2.4. Tribo-Electrification

Tribo-electric charge to mass ratio (*Q*/*M*) was determined using an electrostatic charge measurement apparatus, based on a shaking concept [26]. Powder (~0.1 g) was placed inside a stainless steel cylindrical container (10 mL) and shaken in a horizontal direction (Retsch MM 400) for 0.5, 2, 5 and 10 min at a vibration frequency of 20 Hz. The charged powder particles were then poured into a Faraday cup, connected to an electrometer (Keithley Model 6514). A Faraday cup comprises two concentric cups made up of a conducting material. The outer cup is slightly larger and acts as an electrical shield and a lid covers it. Both are very important to prevent the effect of any extraneous electric fields. The inner cup is directly attached to an electrometer for charge measurement and can be removed to measure the weight of the sample poured. The two cups are separated by a PTFE insulator. As charged samples are loaded into the inner Faraday cup, this induces an equal but opposite charge on the wall of inner faraday cup, providing the net charge on the object [20,28,29]. The resolution of the charge measurement was in nano-Coulombs (nC). The charge to mass ratio (*Q/M*) was calculated by dividing the final charge with the final mass of the respective powder.

Each tribo-electric charging test was repeated three times and the shaking container was cleaned between each test by washing with isopropyl alcohol, rinsing with water and drying with compressed air to remove any residual deposits, impurities and surface charges. All the powder samples were stored overnight at an ambient temperature (18–24 °C) and humidity (RH 36%–44%) for dissipation of tribo-charging. Studies were carried out at an ambient temperature (18–24 °C) and humidity (RH 36%–44%). Maximum charge was gained after shaking for 0.5 min for polymers and 2 min for theophylline and powder blends of polymer/theophylline. Maximum charge acquisition data (Q_max_) are presented as charge to mass ratio (*Q*/*M*) at the end of each tribo-electrification experiment (*n* = 3).

#### 3.2.5. Surface Adhesion

Particle adherence to the surface of the stainless steel container used in the tribo-electrification studies was calculated from mass difference by deducting the final amount recovered (post-shaking and tapping) from the initial amount of sample loaded into the shaking vessel [10,15] and powder mass loss was demonstrated as a percentage (%) of powder adhesion.

#### 3.2.6. Statistical Analysis

Analysis of variance (ANOVA) (confidence limit of *p* < 0.05) was used to investigate the statistical significance of different underlying factors on tribo-electrification and adhesion properties of Methocel^®^:theophylline powder mixtures.

## 4. Conclusions

The study confirms that the particle size, substitution, molecular size and concentration of cellulose ethers all may affect the charging and adhesion behaviour of theophylline. An electrostatic charge-assisted ordering has been showed to be an efficient tool for the dissipation of charge on the API. The charge and adhesion were highly dependent on the concentration, particle size, substitution ratios and molecular size of the cellulose ethers (*p* < 0.05). The decrease in surface adhesion and charge dissipation of theophylline powder mixtures is intuitively expected to further improve processability, which is expected to have a positive effect on the finished pharmaceutical dosage forms.

## Supplementary Materials

Supplementary materials can be accessed at: http://www.mdpi.com/1996-1944/8/4/1482/s1.
