# Supplementary material for: Tribo-electrification and Powder Adhesion Studies in the Development of Polymeric Hydrophilic Drug Matrices
Source: Materials (Basel). 2015 Mar 30;8(4):1482–98. doi: 10.3390/ma8041482 (PMC5507032; doi:10.3390/ma8041482)
Supplement: Supplementary file 1 [file materials-08-01482-s001.pdf]

Supplementary Information

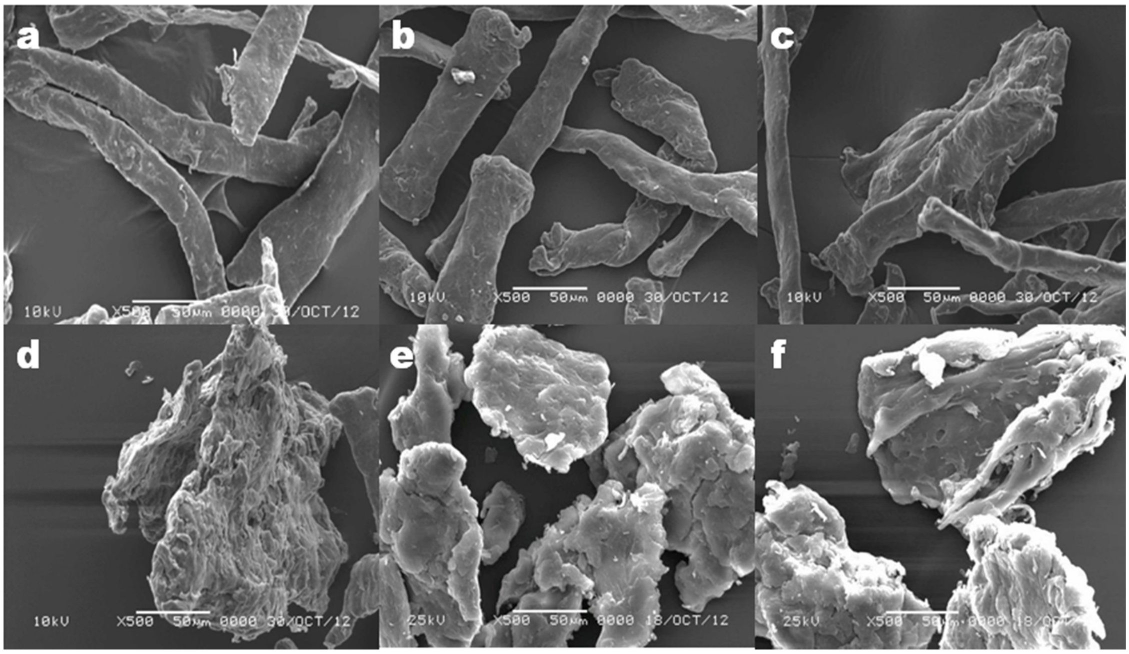

Figure S1. SEM micrographs of different cellulose ethers, (**a**) A4M; (**b**) F4M; (**c**) E4M; (**d**) K4M; (**e**) K15M and (**f**) K100M.

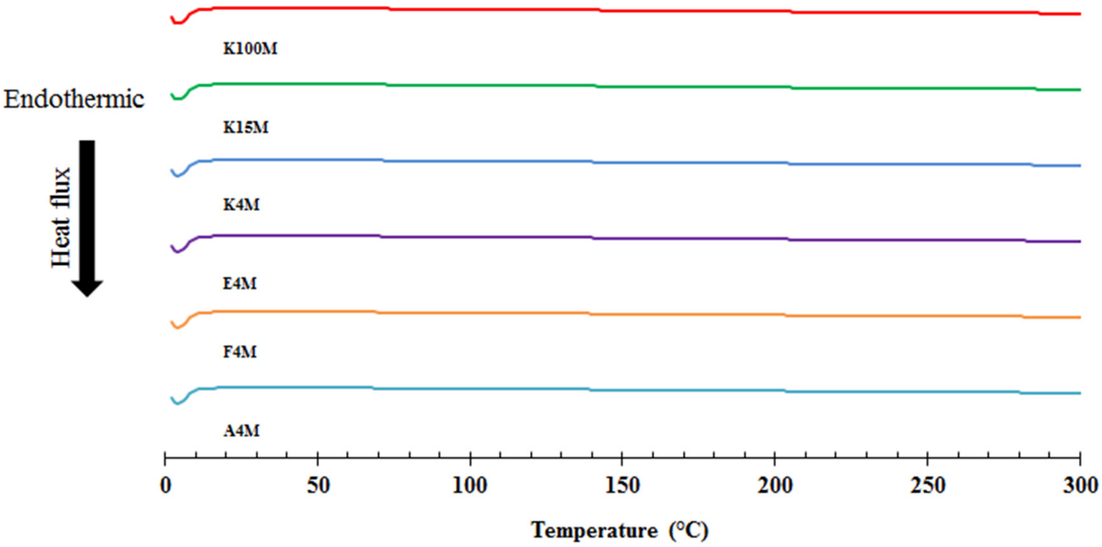

Figure S2. DSC profiles of MC/HPMC.

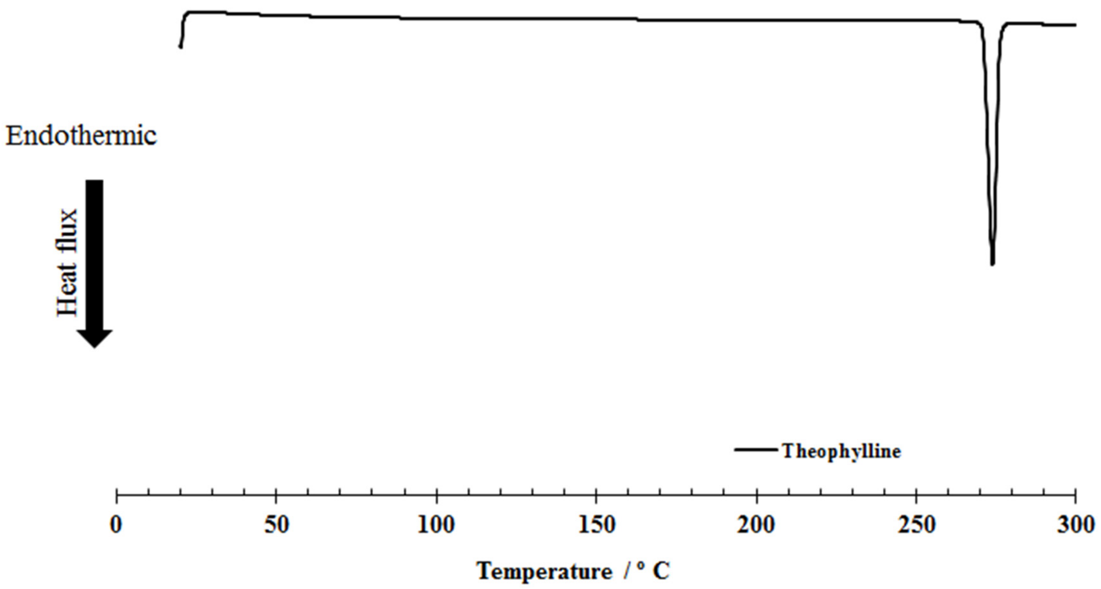

Figure S3. DSC profiles of theophylline.

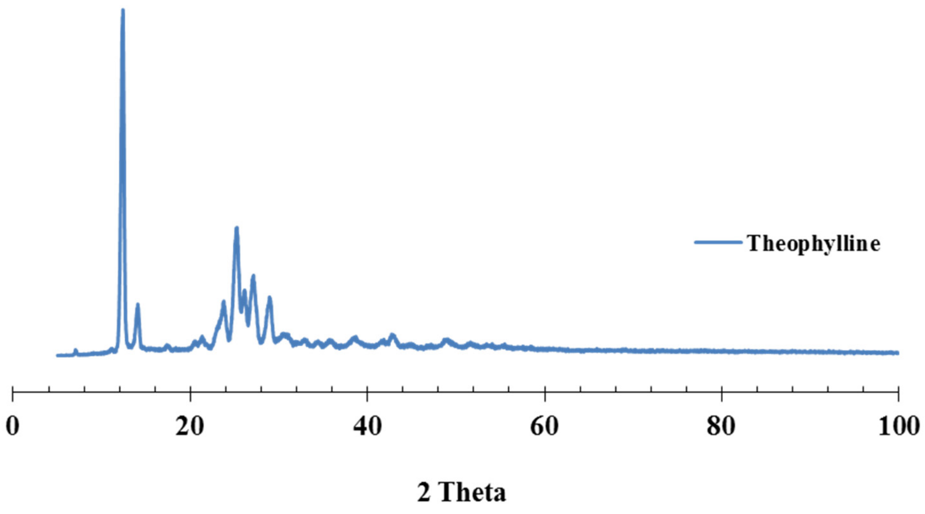

Figure S4. XRD profile theophylline.
